# Operando Raman Spectroscopy Reveals Backpressure‐Controlled Dynamics in Diluted CO_2_ Electrolysis

**DOI:** 10.1002/advs.75908

**Published:** 2026-06-09

**Authors:** Muhammad Adib Abdillah Mahbub, Bashir Eid, Kinran Lau, Nini Zhang, Thomas Quast, Ann Cathrin Brix, Debanjan Das, Wolfgang Schuhmann

**Affiliations:** ^1^ Analytical Chemistry–Center For Electrochemical Sciences (CES) Faculty of Chemistry and Biochemistry Ruhr University Bochum Bochum Germany

**Keywords:** CO_2_, CO_2_ reduction, microenvironment, Raman spectroscopy, water dynamics

## Abstract

Understanding the competition between the electrochemical CO_2_ reduction reaction (eCO_2_RR) and the hydrogen evolution reaction (HER) is crucial for efficient CO_2_ conversion, particularly under limited reactant availability using diluted CO_2_. We investigate the interplay between the operating conditions at various differential backpressures concerning CO_2_ availability, water behavior at the electrified catalyst layer, and how these factors modulate the eCO_2_RR‐to‐formate selectivity and HER activity. We show that H_2_ formation can be suppressed while maintaining high formate selectivity even using highly diluted CO_2_ (2.25%) by increasing the differential backpressure from the gas side of the gas‐diffusion cathode in the flow‐through electrolyzer, which enhances CO_2_ transport to the catalyst layer through reshaping the triple‐phase boundary. Operando Raman spectroscopy reveals the accumulation and perturbation of water on the catalyst layer at negative potentials, accompanied by weakening of the hydrogen‐bond network. Moreover, even higher differential backpressure minimizes carbonate formation, preserves eCO_2_RR kinetics, and promotes the displacement of surface water, which in turn minimizes HER.

## Graphical abstract

High backpressure operation governs the CO_2_ reduction reaction (eCO_2_RR) kinetics and selectivity, as well as the CO_2_ and water dynamics, as revealed through operando Raman spectroelectrochemical measurement. Operating eCO_2_RR near diffusion‐limited conditions provides a platform for investigating the competition between eCO_2_RR and the hydrogen evolution reaction (HER).

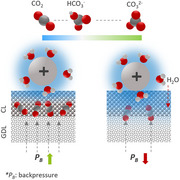

## 1 Introduction

System‐level parameters such as pressure and temperature play a critical role in determining electrochemical CO_2_ reduction reaction (eCO_2_RR) performance [1, 2, 3]. In aqueous CO_2_ electrolysis, a pressurized system not only aims to increase CO_2_ solubility but is also effective for managing water at the electrode interface, where water serves as an abundant proton source and influences the competition between eCO_2_RR and HER. Adjusting a suitable differential backpressure across the catalyst‐modified gas‐diffusion electrode (GDE) can enhance CO_2_ availability at the reaction interface, stabilize CO_2_‐derived intermediates, and promote protonation pathways, thereby steering the selectivity toward hydrogenated products such as formate, acetate, or methane, depending on the nature of the Cu‐based catalyst and the reaction environment [4, 5, 6]. Despite these effects, the mechanistic consequence of differential backpressure on the reaction environment and product selectivity remains to be investigated.

The competition between HER and eCO_2_RR, and importantly also product selectivity, is governed by the local environment within the GDE. The gas feed from the backside of the GDE established a three‐phase interface that facilitates high current densities through enhanced mass transport [7]. However, rapid CO_2_ consumption can induce reactant depletion and the concomitant increase of local alkalinity drives the chemical equilibrium promoting (bi)carbonate formation, ultimately resulting in salt precipitation at the catalyst surface [8, 9, 10]. Operation using diluted CO_2_ resembles a stress‐test condition where low reactant concentration imposes mass‐transport limitation. Hence, it is imperative to maintain sufficient local CO_2_ availability that determines eCO_2_RR kinetics at the reaction sites.

Beyond mass transport consideration, the negatively charged cathode generates a strong electric field that dynamically influences ions and solvent distribution [11, 12]. This microenvironment modulates the interfacial local pH [13, 14] and water dynamics at the surface, which together govern the reaction mechanism [15]. In aqueous eCO_2_RR, where water concentration is high and sufficient to solvate ions, it accumulates at the interface and reorients in response to the applied potential, leading to disruption and reconfiguration of the H‐bond network. Recent models suggested that potential‐driven cation accumulation at the interface strengthens the local electric field, explaining water reorganization due to strong polarization [16]. Moreover, reports show that GDEs modified with organic molecules, coupled with cation effects, facilitate interfacial water disruption and displacement, enabling dehydrated cations to populate the interface and thereby favoring eCO_2_RR over HER [17, 18, 19]. Besides cations being necessary for stabilization of adsorbed CO_2_ and its intermediates, the coexistence of polarized water at the electrode creates competition between eCO_2_RR and HER. These intricacies require strategies to drive charge transfer toward CO_2_ reduction rather than hydrogen evolution.

We investigate the effect of variations of the differential backpressure across the GDE in a flow‐through eCO_2_RR electrolyzer operating with a low CO_2_‐concentration gas feed using a bismuth‐methylimidazole (Bi[2‐MeIm]) catalyst. This catalyst has previously demonstrated high current density approaching mass‐transport‐limited conditions and high selectivity for formate even at 2.25% CO_2_ concentration [20]. Applying a differential backpressure between the gas and cathode compartments across the GDE modulates the triple‐phase boundary (TPB) by increasing the CO_2_ availability at the catalyst layer. Operando Raman spectroelectrochemical measurements revealed on the one hand that the bicarbonate‐carbonate equilibrium is a descriptor of CO_2_ availability, while on the other hand, an increased backpressure induced a displacement of water, thereby promoting high formate selectivity and suppressing hydrogen evolution.

## 2 Result and Discussion

**FIGURE 1:**
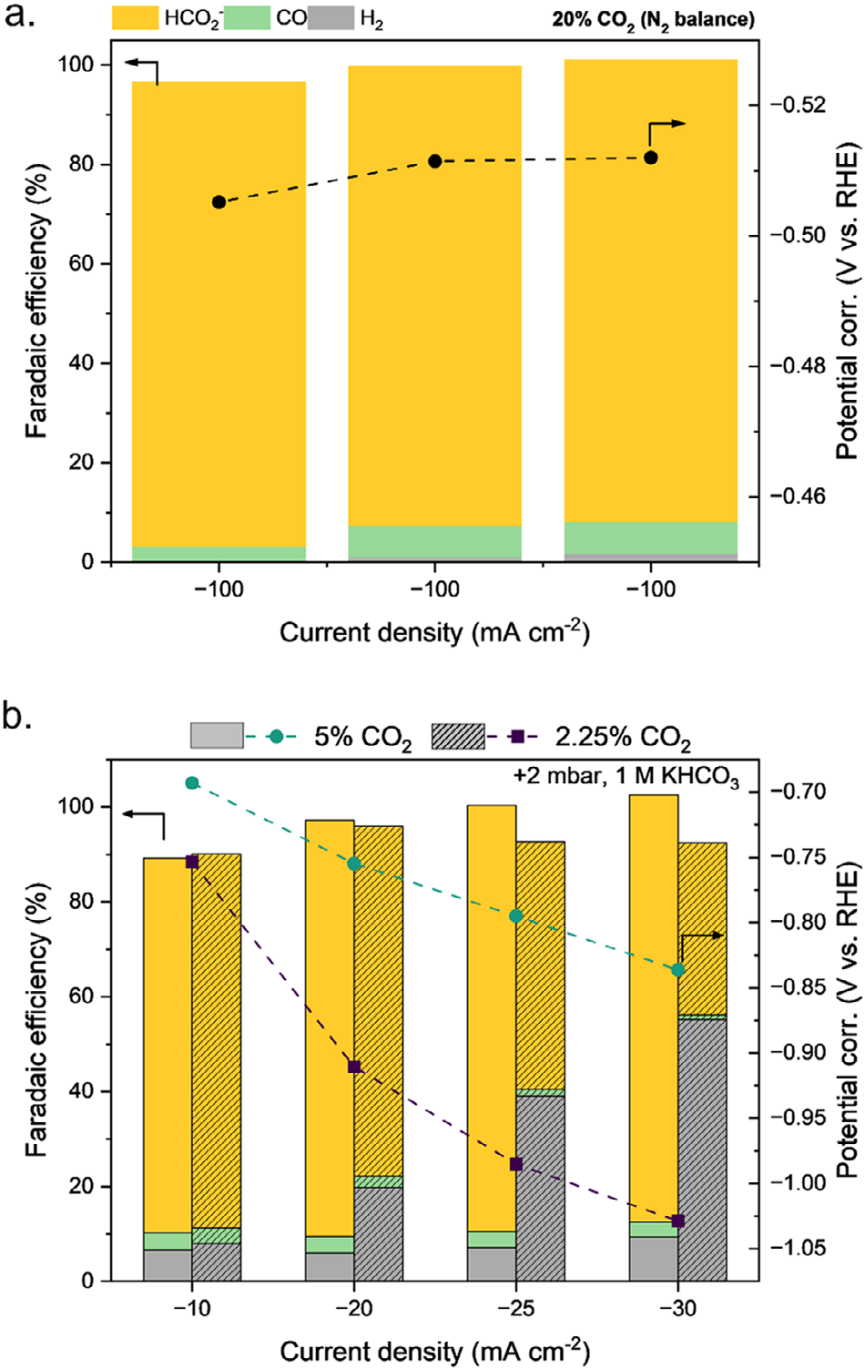
(a) Faradaic efficiencies for formate, CO, and H_2_ at −100 mA cm^−2^ at 20% CO_2_ concentration (N_2_ balance) using a Bi[2‐MeIm]‐modified GDE. (b) Faradaic efficiencies and corrected potentials (V_RHE_) as a function of current densities of Bi[2‐MeIm] at 5% and 2.25% CO_2_ concentration (N_2_ balance).

eCO_2_RR to formate on a Bi[2‐MeIm] catalyst modified GDE using diluted CO_2_ was evaluated in a three‐compartment flow‐through model electrolyzer. The cathode exhibited a geometric area of 0.95 cm^2^, the catholyte compartment had a thickness of 5 mm, Nafion 117 was used as a membrane to prevent formate crossover to the anolyte compartment, Ni‐foam was used as anode, and a Ag/AgCl (3 M KCl) reference electrode was positioned close to the GDE in 1 M KHCO_3_ and 1 M KOH as catholyte and anolyte, respectively. We rule out cation effects by using exclusively K^+^‐based electrolytes with the same concentration in both compartments. The catalyst performance and its pre‐ and post‐electrolysis characterization have been reported earlier, with the catalyst operating at a current density of ‐1 A cm^−2^ while revealing a dynamic transformation during electrolysis under formation of Bi_2_O_2_CO_3_, Bi_2_O_3_, and metallic Bi [20]. Highly active catalysts are required to evaluate the performance using diluted CO_2,_ which was obtained by diluting CO_2_ with N_2_ [21]. We started with 20% CO_2_ concentration with N_2_ balance (total reactant gas flow rate ∼20 mL min^−1^) and applied a constant current density of −100 mA cm^−2^ demonstrating the expected high selectivity of formate generation. (Figure 1a). An initial electroreduction step was applied for pre‐catalyst transformation, followed by chronopotentiometric measurements (Figure S1). At this comparatively high current density, the Bi[2‐MeIm] catalyst showed a constant high selectivity toward formate at a low overpotential of −0.51 V vs. reversible hydrogen electrode (RHE, V_RHE_). At even more diluted CO_2_ concentrations of 5% and 2.25% CO_2_ in the gas feed and operation at lower current densities (Figure 1b), the diminished availability of CO_2_ is substantially modifying the local reaction environment within the GDE.

While the formate selectivity is maintained when operating at 5% CO_2_, HER started to rise at 2.25% CO_2,_ and the reaction kinetics were mainly affected by the mass transport of CO_2_ using the same gas flow rate and backpressure during measurement. When operated at 2.25% CO_2,_ a more cathodic potential is required to reach the applied current density, which leads to a comparatively high $FE_{H_{2}}$. This promotes a more pronounced alkalinization, considering that CO_2_‐to‐HCOO^−^ produces 1 mol of OH^−^ per 2 moles of transferred e^−^, while HER produces 2 moles of OH^−^ [22]. Moreover, the use of bicarbonate electrolytes might contribute to formate formation, due to the chemical equilibrium between CO_2_ and HCO_3_^−^ [23, 24]. This concept is the basis for an electrolyzer fed with liquid bicarbonate rather than gaseous CO_2,_ which converts HCO_3_^−^ into dissolved CO_2_ [25, 26, 27, 28]. Bicarbonate plays multiple roles, not only assisting CO_2_ electrolysis as a pH buffer, but also serving as a proton donor and CO_2_ source [29, 30, 31, 32]. To confirm the contribution of HCO_3_^−^ as a CO_2_ source, and to ensure that most products are derived from low‐concentration CO_2_, we performed a control experiment without 2.25% CO_2_ in the gas feed, using 1 M KHCO_3_ as the catholyte and omitting N_2_ purging in the reservoir (Figure S2a). By this, the removal of in situ generated CO_2_ is prevented and allows us to isolate formate as the only detectable product. Previous reports have shown that neither N_2_‐ nor CO_2_‐purging in the electrolyte affects the CO_2_RR selectivity in a highly concentrated 3 M HCO_3_^−^ as electrolyte. However, when operated at lower concentrations of 0.5 to 2 M KHCO_3_, the CO_2_‐purged solution yields substantially more eCO_2_RR products, suggesting that the dissolved CO_2_ in such a buffer system contributed as an active reactant [23]. The recorded potential and the uncompensated electrolyte resistance derived from the Nyquist plots were used to correct the potential. The more negative potential values and the trace amounts of detected formate with increasing current densities (Figure S2b–f) reflect the minimal contribution of KHCO_3_ as a CO_2_ source. We conclude that the majority of formate produced in our CO_2_ electrolyzer is derived from the gaseous CO_2_. We previously demonstrated that combining a highly active catalyst with a buffered electrolyte can sustain the eCO_2_RR at diluted CO_2_ conditions. However, mass transport limitation remains a critical challenge, particularly at even lower CO_2_ concentrations. While gas diffusion electrodes and flow‐by configuration experiments are commonly employed to improve gas accessibility, they do not guarantee sustained eCO_2_RR if the CO_2_ availability is extremely limited. Under such conditions, the competition of HER dominates the reaction, which makes understanding of the HER mechanism essential, particularly in HCO_3_^−^‐based electrolytes that can act as a proton donor. Electrokinetic analysis and complementary spectroscopic studies indicate that as the applied potential becomes more negative, the primary proton donor for HER shifts from HCO_3_^−^ to H_2_O [33, 34].

Operating eCO_2_RR with diluted CO_2_ drives the potential more cathodically, thus increasing HER competition by water reduction. At these high cathodic potentials, a high electric field is established, causing the accumulation of water and ions at the electrified surface. Therefore, modulating the microenvironment of the catalyst surface and thereby controlling the water population at the interface is critical. Besides suppressing HER, boosting eCO_2_RR kinetics by enriching CO_2_ availability at the catalyst layer is essential for sustaining the reaction.

We hence investigated the effect of differential backpressures between the gas and catholyte compartments at the GDE with respect to the local CO_2_ availability at the catalyst. We hypothesize that H_2_O and CO_2_ compete for the same or nearby adsorption sites on the catalyst surface, and hence a higher CO_2_ concentration promotes eCO_2_RR over HER.

**FIGURE 2:**
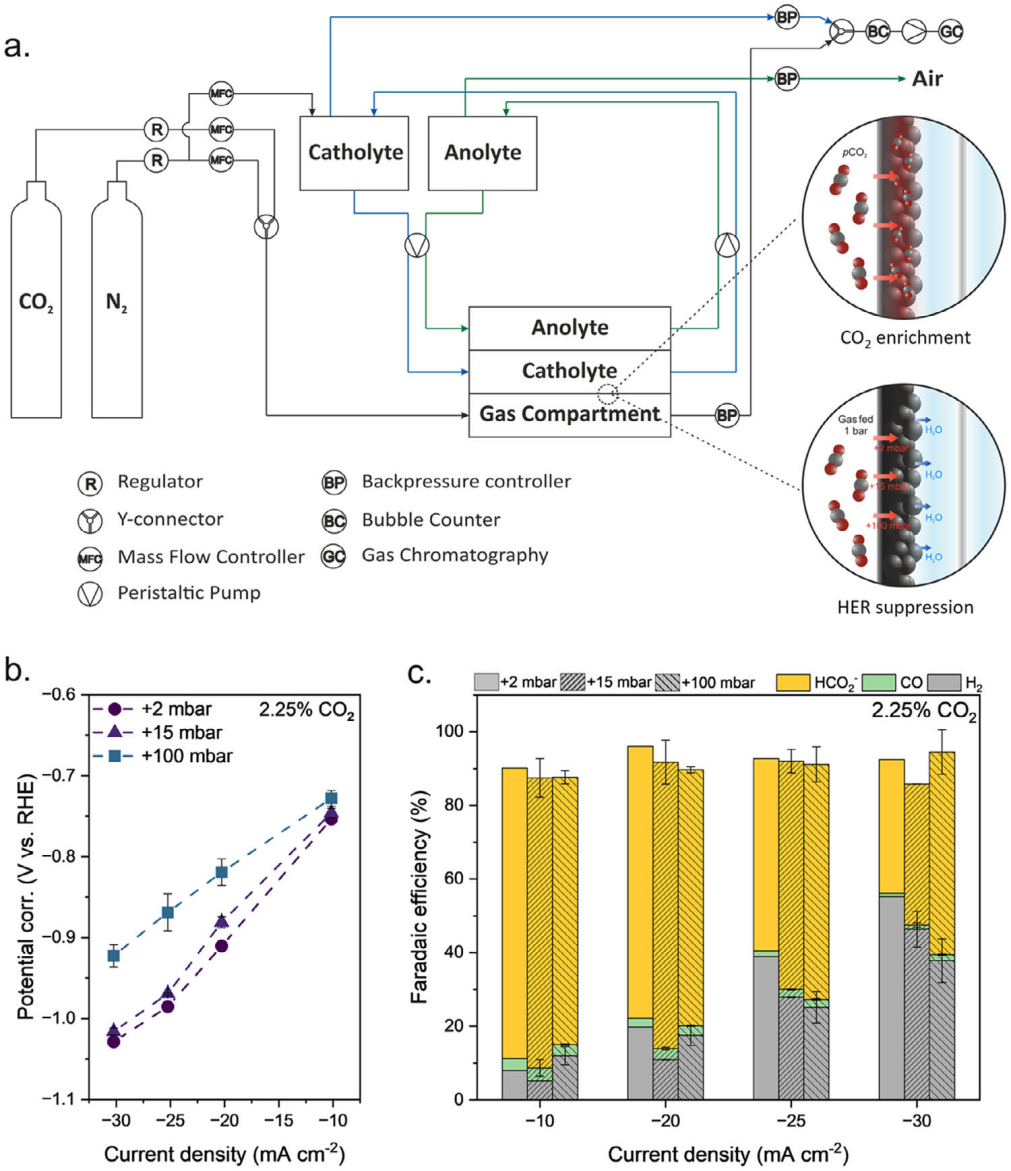
(a) Experimental setup with controllable differential backpressure across the GDE and illustration of the effect of applying higher backpressure between the gas and catholyte compartments. eCO_2_RR performance of (b) current density vs. corrected potentials (V_RHE_), and (c) Faradaic efficiency at diluted CO_2_ of 2.25% with various differential backpressures.

The experiment utilized a setup with a gas feed of 2.25% CO_2_ at a regulated inlet pressure of 1 bar. Various backpressures (+2, +15, and +100 mbar) were applied across the GDE (Figure 2a). The differential backpressure was controlled using a water column positioned in the outlet stream between the gas compartment and the bubble counter, while a second water column (1 mbar) was placed into the catholyte reservoir outlet. Precautions were taken to ensure that no gas bubbles formed or adhered to the electrode in the catholyte compartment during increasing the pressure, as they could otherwise cause electrical insulation or disrupt electrochemical performance. Given that the setup was a lab‐scale model flow‐through electrolyzer cell with an electrolyte channel height of 1.1 cm, the internal hydrostatic pressure gradient was deemed negligible. A uniform distribution of CO_2_ across the GDE was obtained, allowing for deriving the effect of the differential pressure modulation on the microenvironment at the TPB of the GDE.

During eCO_2_RR at −10 mA cm^−2^, both the recorded overpotentials and formate selectivity remain largely unchanged for all pressure conditions (Figure 2b,c). This indicates that the applied currents remain within the limits of sufficient CO_2_ availability without severe mass transport constraints. Conversely, at higher current densities, applying an elevated backpressure of +100 mbar reduces overpotentials, suggesting improved energy efficiency due to the enhanced CO_2_ availability in the catalyst layer. HER decreases moderately with increasing backpressure (Figure 2c), likely due to the differential pressure‐driven displacement of surface water. Furthermore, control experiments at low (+2 mbar) and high (+50 mbar) backpressure were conducted using 100% CO_2_ gas at current densities of up to −100 mA cm^−2^ (Figure S3). The results confirmed that for highly available CO_2_, the eCO_2_RR performance remains unaffected by backpressure. Increasing the differential pressure across the GDE is linearly correlated with improved CO_2_ and is expected to move the TPB deeper into the catalyst layer [35, 36]. Similarly, a high backpressure (+20 mbar) can mitigate catalyst perspiration while maintaining eCO_2_RR selectivity [37]. Although operating at low CO_2_ concentration inevitably increases overpotential, this effect is attributed to electrocapillary‐driven electrowetting, which is highly sensitive to the potential [38]. Therefore, maintaining a modest differential backpressure is crucial for preserving the TPB and preventing severe flooding of the catalyst layer, particularly when using a flexible carbon paper substrate with crack‐free micropores. Motivated by these findings, we investigated the effect of the differential backpressure on the local interfacial environment using operando Raman spectroelectrochemistry. We designed a GDE‐based Raman cell with separated catholyte and anolyte compartments (Figure S4) to perform measurements that mimic the experiments in the flow‐through electrolyzer. Bi[2‐MeIm] catalysts were interrogated with and without electrolyte (Figure S5) [20, 39].

A significant transformation toward Bi_2_O_2_CO_3_ was observed, which is known as the active phase for CO_2_RR to formate, indicated by two bands related to Bi‐O at 158 cm^−1^ and the CO_3_^2−^ band at 1066 cm^−1^. Upon introducing the electrolyte, a HCO_3_^−^ band appeared at 1017 cm^−1^. The mechanism is consistent with the formation of Bi_2_O_2_CO_3_ nanosheets, which consist of [Bi_2_O_2_]^2+^ layers with sandwiched CO_3_^2−^ [40]. The intense Raman bands are attributed to the high concentration of bicarbonate electrolyte, which facilitates the formation of thicker Bi_2_O_2_CO_3_ nanosheets [41].

The influence of backpressure on the dynamic surface species and the water population was evaluated using operando Raman spectroelectrochemistry. A set of experiments was conducted using a Bi[2‐MeIm] catalyst‐modified GDE supplied with a continuous 2.25% CO_2_ flow (20 mL min^−1^) in 1 M KHCO_3_ as catholyte (non‐CO_2_‐saturated, pH = 8.8). This configuration was chosen to closely replicate the electrolyzer experiments, in which the potential was scanned from −0.9 to −2.3 V vs. Ag/AgCl (3 M KCl) at different backpressures (+2 and +15 mbar). All spectra (Figure S6) were treated using the same smoothing, baseline correction, and normalization procedures. The applied potential is further reported in a reversible hydrogen electrode (V_RHE_) for reliable comparison with experiments in a flow cell.

**FIGURE 3:**
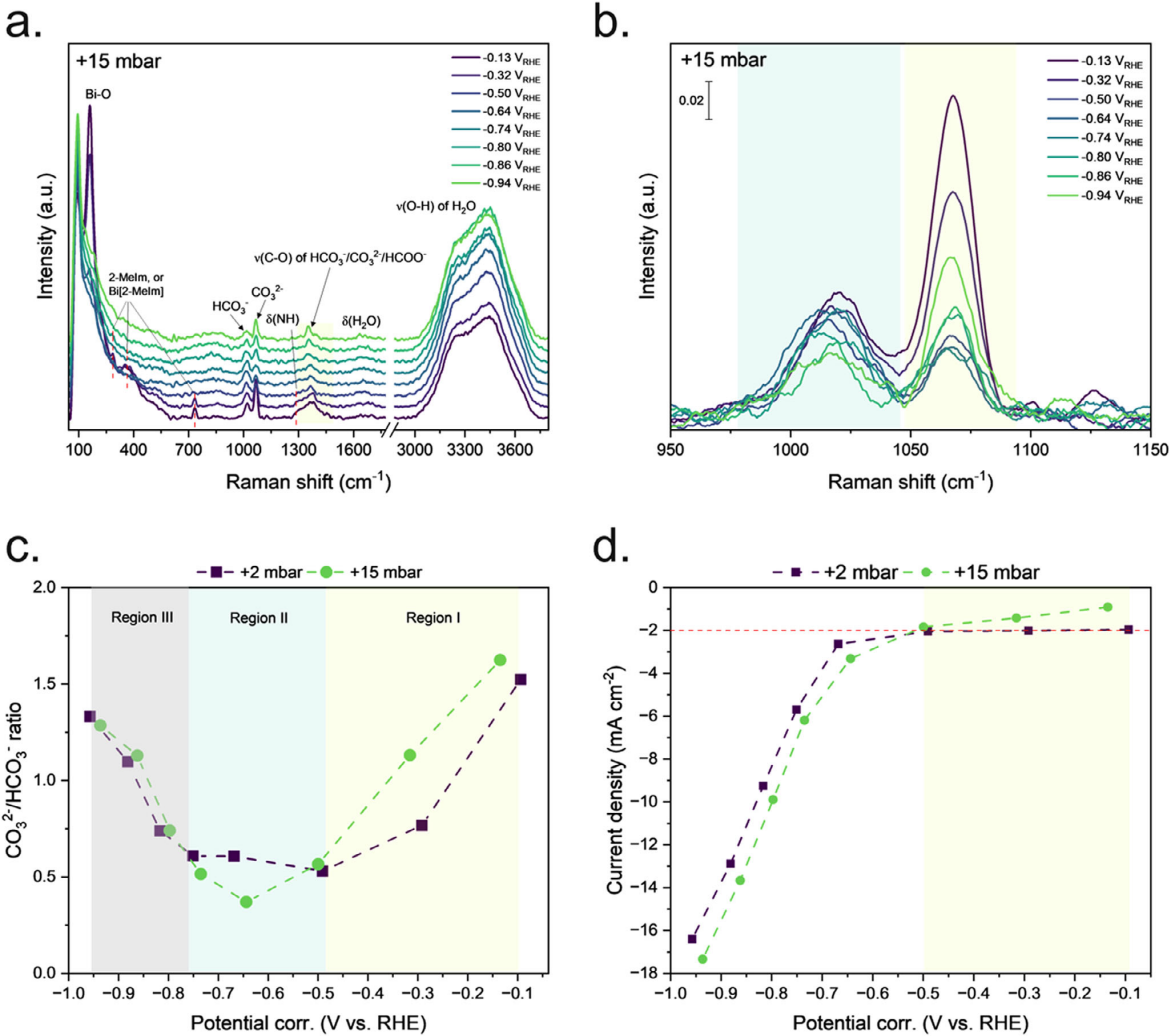
Operando Raman spectroelectrochemistry in 1 M KHCO_3_ at 2.25% CO_2_ concentration and +15 mbar backpressure. (a) Normalized potential‐dependent Raman spectra, and (b) enlarged bicarbonate and carbonate regime. (c) Quantitative analysis of the carbonate‐bicarbonate ratio as a function of the corrected potentials (V_RHE_). (d) Corrected potential‐current density plot corresponding to −0.9 to −2.3 V vs. Ag/AgCl (3 M KCl).

Figure 3a and Figure S7a show characteristic bands in the post‐electrolysis spectra, including Bi‐O and CO_3_^2−^ bands, which exhibit intense features because of electrolyte‐mediated catalyst transformation, as well as bands associated with HCO_3_^−^, the H_2_O bending vibration mode at 1640 cm^−1^, and the water (OH) stretching vibration mode at 3000–3800 cm^−1^ from the electrolyte. Notably, both Bi‐O and CO_3_^2−^ decrease concurrently due to catalyst reduction when the potential is scanned from −0.1 to −0.5 V_RHE_. At more cathodic potentials, OH^−^ is generated via eCO_2_RR and HER, leading to the accumulation of (bi)carbonate ions at the electrode surface. Figure 3b and Figure S7b show enlarged spectra of the HCO_3_^−^ and CO_3_^2−^ bands, which were used to monitor changes in local alkalinity arising from the backpressure‐controlled (bi) carbonate equilibria. Following catalyst reduction, the CO_3_^2−^ band becomes more pronounced at more negative potentials, whereas the HCO_3_^−^ band is slightly decreased.

The CO_3_^2−^/HCO_3_^−^ intensity ratio (Figure 3c) was plotted as a descriptor of local CO_2_ availability and its dependence on the backpressure (see Equations S4–S9 for the relevant reaction pathways). At low applied potentials (−0.1 and −0.5 V_RHE_, region I), a high CO_3_^2−^/HCO_3_^−^ ratio is observed, which is primarily attributed to the strong bismuth oxycarbonate (Bi_2_O_2_CO_3_) contribution. However, this feature originates from catalyst transformation rather than from the (bi)carbonate equilibrium. Therefore, this potential region is further excluded from the discussion. In region II (−0.5 to −0.75 V_RHE_), the CO_3_^2−^/HCO_3_^−^ ratio reaches a plateau at +2 mbar, whereas the ratio is lower at a backpressure of +15 mbar, indicating suppressed carbonate formation. The enrichment of CO_2_ in the GDE shifts the equilibrium to the HCO_3_^−^/CO_2_ side and mitigates conversion to CO_3_^2−^, which also facilitates catalyst transformation to Bi_2_O_2_CO_3_. Given the buffering capacity of the 1 M KHCO_3_ electrolyte, applying higher backpressures maintains eCO_2_RR by keeping the CO_2_ reactant available. We therefore assign this region as CO_2_RR‐dominated at high backpressure with formate as the primary product. While this ratio can be used to determine surface pH, which alters eCO_2_RR selectivity, we emphasize that carbonate formation driven by excess OH^−^ leads to CO_2_ depletion, which decreases eCO_2_RR performance. Mechanistically, formate production generates one mole of OH^−^ per mole of product, while HER produces two moles of OH^−^. Consequently, the lower CO_3_^2−^/HCO_3_^−^ ratio observed at higher backpressure signifies the formate‐selective pathway, characterized by lower OH^−^ generation, less carbonate formation, and preserving CO_2_ as an active reactant.

The current density (normalized by the geometric area) and the corresponding Nyquist plot for correction of the uncompensated resistance in the potential range from −0.9 to −2.3 V vs. Ag/AgCl (3 M KCl) are provided in Figure S8. A potential correction to the reversible hydrogen electrode was necessary due to differences between the Raman cell and the flow cell geometry. The corrected potential‐current density plot (Figure 3d) shows slightly higher current densities at elevated backpressure. If the reaction mechanism at both backpressure conditions is similar, the increased current leads to increased stoichiometric OH^−^ generation, and thus a higher CO_3_^2−^/HCO_3_^−^ ratio due to carbonate formation. However, the lower CO_3_^2−^/HCO_3_^−^ ratio at high backpressure further confirms the formate‐selective eCO_2_RR pathway. To further validate the effect of stoichiometric OH^−^ formation, an additional experiment was performed at low current densities (−3 to −20 mA cm^−2^, Figure S9). The result reveals a consistently lower CO_3_^2−^/HCO_3_^−^ ratio and a lower operating potential (Figure S10) at higher backpressure.

To support the assignment of formate‐related species, control Raman experiments were conducted (Figure S11) using a potassium formate (1 M KHCO_2_) solution. Characteristic formate bands at 1355 and 1385 cm^−1^ were identified, which overlap with the ν(C‐O) vibrational modes of HCO_3_^−^ and CO_3_^2−^. In the operando measurements, the formate concentration is expected to be low due to the use of a 2.25% CO_2_ feed and the presence of a strong bicarbonate electrolyte. The C‐H vibration band of the HCOO^−^ species was also not detected. By shifting to more negative potentials (−0.75 to −0.95 V_RHE_, region III), the CO_3_^2−^/HCO_3_^−^ ratio increased for both backpressure conditions, indicating a shift in the equilibrium toward carbonate formation, suggesting substantial OH^−^ generation, due to HER becoming the dominant reaction pathway.

The water (O‐H) stretching band in the Raman spectra (3000–3800 cm^−1^) is a commonly used descriptor for probing H‐bond strength and the population of water at surfaces. This broad spectral feature originates from the intramolecular coupling between the O‐H stretching vibration mode and an H_2_O bending overtone due to Fermi resonance [42]. Although analysis of the H_2_O bending vibration mode can provide a more direct and exclusive picture of the water structure [43], the broad O‐H stretching vibration in the KHCO_3_‐in‐water system is plausibly dominated by contributions from bulk and interfacial water. The water (O‐H) stretching mode displayed comparable intensities at both backpressure conditions in the presence of the electrolyte (Figure S12). Therefore, any subsequent changes in this band with applied potential can be used as a probe to evaluate potential‐dependent water dynamics.

**FIGURE 4:**
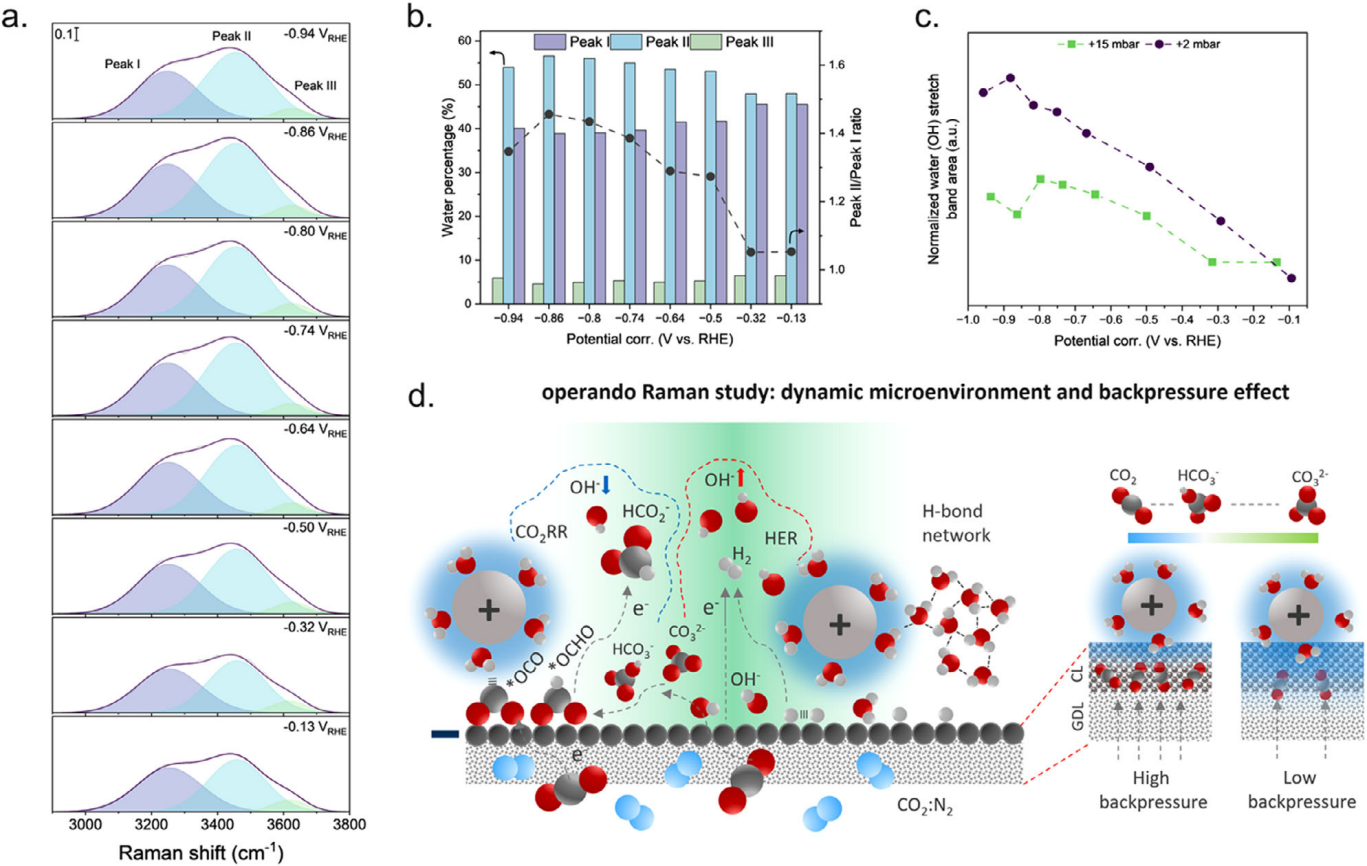
(a) Normalized and deconvoluted Raman spectra. (b) Water percentage and the corresponding peak I/peak II ratio derived from the O‐H stretching vibration signal at various potentials and +15 mbar backpressure. (c) Water (O‐H) stretching band area at +2 and +15 mbar backpressures. (d) Illustration of a dynamic microenvironment (left side) involving eCO_2_RR and HER, carbonate‐bicarbonate formation, the H‐bond network on an electrified surface, and the effect of backpressure on CO_2_ enrichment and displacement of water (right side).

Deconvolution of the water (O‐H) stretching vibration mode (Figure 4a and Figure S13a) reveals two dominant components (peak I and II centered at ∼3250 and ∼3455 cm^−1^, respectively), along with a minor high‐frequency component (peak III) at ∼3620 cm^−1^. Upon increasing cathodic potentials, the intensities of peaks I and II increase in a nonlinear manner. However, the relative contribution of peak I decreases, while peak II becomes more pronounced, and peak III remains steady (Figure 4b and Figure S13b). Accordingly, the peak II/peak I ratio increases with more negative potentials. This trend reflects a progressive weakening of the H‐bonds, as higher‐frequency O‐H stretching vibration modes are associated with less strongly H‐bonded water molecules [42]. On this basis, the deconvoluted O‐H stretching bands are assigned to distinct water structures with different H‐bond configurations, ranging from a strengthened H‐bond at low frequency to a weakened H‐bond at higher frequencies. We point out that the weakened H‐bond is primarily driven by potential‐dependent effects, as more cations accumulate and disrupt the water H‐bond network at more cathodic potentials. Concurrently, a cumulative increase in water population is also observed as the potential becomes more negative, indicating potential‐driven water accumulation. In contrast, varying the applied backpressure does not significantly alter the speciation of the water structure. Such changes in water structure are more commonly observed in studies addressing cation effects involving structure‐maker and structure‐breaker cations [44], electrolyte‐concentration‐driven disordering of the H‐bond network [45, 46], and in variations in water solvation dynamics induced by different solvents [47, 48]. By comparison, our approach demonstrates that applying backpressure affects the overall water population rather than its structural speciation, with higher backpressure leading to a depletion in water content (Figure 4c). This trend aligns with the electrochemical measurement, in which increased backpressure correlated with higher formate selectivity and low HER. Due to the sensitivity of the GDE‐based Raman measurements with respect to gas‐diffusion layer (GDL) quality and gas flow rate variations, an additional set of experiments with +2 and +15 mbar backpressure was performed to show reproducibility (Figures S14 and S15), and the results were consistent with the initial measurements.

Our findings indicate that backpressure dynamically controls the local environments (Figure 4d), providing a means to study the competition of eCO_2_RR and HER under CO_2_‐limited conditions. While our previous work on Ag‐based GDEs offered insight into the complex competition [49], the electrolyzer experiments complemented with Raman spectroelectrochemistry enable us to understand how CO_2_ selectivity and HER suppression are governed by backpressure and applied potential. At low back pressure, limited CO_2_ availability, and enhanced OH^−^ generation promote carbonate formation, thereby favoring the HER. In contrast, elevated backpressure enriches CO_2_ in the GDE, shifting the (bi)carbonate equilibrium toward a lower CO_3_^2−^/HCO_3_^−^ ratio. A substantial CO_2_ enrichment at a high backpressure might partially displace surface water, leading to suppression of HER, while the applied potential alone does not alter water speciation. A similar case has been reported for the competition between adsorbed *CO and water, where depletion of the O‐H stretching mode intensity was attributed to the displacement of interfacial water [50].

Importantly, the displacement of water does not imply complete removal of water from the interface, as the O‐H stretch band remains detectable and protons from water are still required for the reaction. Rather, we can speculate that there is a competition for adsorption sites between *CO_2_ and *H_2_O, and the balance between these species ultimately dictates eCO_2_RR selectivity. Notably, this backpressure effect is even clearly observed when operating the eCO_2_RR at low CO_2_ concentration approaching diffusion limitation. A comparable observation has been reported for organic film‐modified electrodes operating at low CO_2_ conditions, where maintaining the local CO_2_/H_2_O concentration enabled sustained eCO_2_RR even at low CO_2_ partial pressure [51]. Similarly, recent operando Raman investigation of eCO_2_RR to ethylene at extremely high pressure (20 bar) demonstrated enhanced CO_2_ and HCO_3_^−^ bands accompanied by depletion of the CO_3_^2−^ signal [35]. The bicarbonate species might shift deeper to the catalyst layer‐membrane interface upon applying a high pressure to the membrane electrode assembly (MEA). Our finding complements this interpretation by suggesting that bicarbonate shifting is accompanied by the displacement of water at the catalyst layer. Finally, applying high pressure has a broader significance, particularly for larger‐scale CO_2_ electrolyzers where spatial distribution becomes a major issue [52]. Adjusting the gas pressure not only sustains CO_2_ availability but also mitigates inhomogeneity of the reactant over the GDE, thereby stabilizing current density and maintaining uniform product selectivity.

## 3 Conclusion

Our conceptual work revealed how backpressure across the GDE can influence eCO_2_RR kinetics and its selectivity to formate while simultaneously impeding HER. Operando Raman spectroelectrochemistry demonstrates that elevated backpressure enhances local CO_2_ availability, suppresses carbonate formation, and promotes the reaction toward eCO_2_RR products. Experiments conducted under diluted CO_2_ conditions further elucidate the interplay between CO_2_ availability, water dynamics, and HER competition, highlighting the sensitivity of the selectivity to local mass transport limitations. Despite the tendency of HER to dominate at diffusion‐limited conditions, regulating gas‐to‐liquid pressure plays a critical role in maintaining eCO_2_RR performance. While the applied potential perturbs the H‐bond network, applying higher pressure modulates the water population. These insights provide a framework for pressure‐controlled operation in advanced electrolyzer configurations, such as large‐scale MEA electrolyzers, to control the spatial distribution of CO_2_ and to balance the CO_2_/H_2_O ratio at the GDE‐membrane interface.

## Author Contributions

**Muhammad Adib Abdillah Mahbub**: conceptualization, investigation, writing – original draft, writing – review and editing, visualization, methodology, formal analysis. **Thomas Quast**: methodology. **Kinran Lau**: formal analysis, investigation, writing – review and editing. **Ann Cathrin Brix**: methodology, writing – review and editing. **Wolfgang Schuhmann**: funding acquisition, writing – review and editing, project administration, supervision, resources, conceptualization. **Nini Zhang**: methodology, investigation, writing – review and editing. **Debanjan Das**: formal analysis. **Bashir Eid**: methodology, investigation.

## Funding

European Research Council (ERC) under the European Union's Horizon 2020 research and innovation programme (grant agreement CasCat [833408])

## Conflicts of Interest

The authors declare no conflicts of interest.

## Supporting information




**Supporting File**: advs75908‐sup‐0001‐SuppMat.docx.

## Data Availability

The data that support the findings of this study are available from the corresponding author upon reasonable request.

## References

[1] R. Küngas , “Review—Electrochemical CO_2_ Reduction for CO Production: Comparison of Low‐ and High‐Temperature Electrolysis Technologies,” Journal of the Electrochemical Society 167, no. 4 (2020): 44508, 10.1149/1945-7111/ab7099.

[2] Y. Li , H. Liu , J. Raj , M. Pishnamazi , and J. Wu , “Elevated Temperature and Pressure Driven Ampere‐level CO_2_ Electroreduction to CO in a Membrane Electrode Assembly Electrolyzer,” EES Catalysis 3 (2025): 843–855, 10.1039/D5EY00034C.

[3] J.‐W. Hurkmans , H. M. Pelzer , T. Burdyny , J. Peeters , and D. A. Vermaas , “Heating Dictates the Scalability of CO_2_ Electrolyzer Types,” EES Catalysis 3 (2025): 305–317, 10.1039/D4EY00190G. 39802814 PMC11721209

[4] L. Huang , G. Gao , C. Yang , et al., “Pressure Dependence in Aqueous‐based Electrochemical CO_2_ Reduction,” Nature Communications 14, no. 1 (2023): 2958, 10.1038/s41467-023-38775-0. PMC10205702 37221228

[5] J. Li , Y. Kuang , X. Zhang , et al., “Electrochemical Acetate Production from High‐pressure Gaseous and Liquid CO_2_,′,” Nature Catalysis 6, no. 12 (2023): 1151, 10.1038/s41929-023-01046-8.

[6] H. Wu , B. Tian , W. Xu , et al., “Pressure‐Dependent CO_2_ Electroreduction to Methane over Asymmetric Cu‐N_2_ Single‐Atom Sites,” Journal of the American Chemical Society 146, no. 32 (2024): 22266, 10.1021/jacs.4c04031. 38996381

[7] X. Wang , C. Tomon , T. Bobrowski , et al., “Gaining the Freedom of Scalable Gas Diffusion Electrodes for the CO_2_ Reduction Reaction,” ChemElectroChem 9, no. 21 (2022): 202200675, 10.1002/celc.202200675. PMC9828112 36636096

[8] A. Goyal , G. Marcandalli , V. A. Mints , and M. T. M. Koper , “Competition between CO_2_ Reduction and Hydrogen Evolution on a Gold Electrode under Well‐Defined Mass Transport Conditions,” Journal of the American Chemical Society 142, no. 9 (2020): 4154–4161, 10.1021/jacs.9b10061. 32041410 PMC7059182

[9] J. A. Rabinowitz and M. W. Kanan , “The Future of Low‐temperature Carbon Dioxide Electrolysis Depends on Solving One Basic Problem,” Nature Communications 11, no. 1 (2020): 5231, 10.1038/s41467-020-19135-8. PMC7567821 33067444

[10] M. Sassenburg , M. Kelly , S. Subramanian , W. A. Smith , and T. Burdyny , “Zero‐Gap Electrochemical CO_2_ Reduction Cells: Challenges and Operational Strategies for Prevention of Salt Precipitation,” ACS Energy Letters 8, no. 1 (2023): 321–331, 10.1021/acsenergylett.2c01885. 36660368 PMC9841607

[11] L. D. Chen , M. Urushihara , K. Chan , and J. K. Nørskov , “Electric Field Effects in Electrochemical CO_2_ Reduction,” ACS Catalysis 6, no. 10 (2016): 7133–7139, 10.1021/acscatal.6b02299.

[12] X. Tian , A. Tosello Gardini , U. Raucci , H. Xiao , Y. Zhuo , and M. Parrinello , “Electrochemical Potential‐driven Water Dynamics Control CO_2_ Electroreduction at the Ag/H_2_O Interface,” Nature Communications 16, no. 1 (2025): 10636, 10.1038/s41467-025-65630-1. PMC12660881 41309570

[13] S. Dieckhöfer , D. Öhl , J. R. C. Junqueira , T. Quast , T. Turek , and W. Schuhmann , “Probing the Local Reaction Environment during High Turnover Carbon Dioxide Reduction with Ag‐Based Gas Diffusion Electrodes,” Chemistry—A European Journal 27, no. 19 (2021): 5906–5912, 10.1002/chem.202100387. 33527522 PMC8048634

[14] O. Ayemoba and A. Cuesta , “Spectroscopic Evidence of Size‐Dependent Buffering of Interfacial pH by Cation Hydrolysis during CO_2_ Electroreduction,” ACS Applied Materials & Interfaces 9, no. 33 (2017): 27377–27382, 10.1021/acsami.7b07351. 28796478

[15] Y.‐H. Wang , S. Zheng , and W.‐M. Yang , “In Situ Raman Spectroscopy Reveals the Structure and Dissociation of Interfacial Water,” Nature 600 (2021): 81–85, 10.1038/s41586-021-04068-z. 34853456

[16] A. J. King , J. C. Bui , A. Z. Weber , and A. T. Bell , “Revealing the Role of the Electrical Double Layer in Electrochemical CO_2_ Reduction,” ACS Catalysis 15, no. 17 (2025): 14588–14600, 10.1021/acscatal.5c03725.

[17] M. Chang , S. Yoo , W. Ma , H. Girault , Y. J. Hwang , and X. Hu , “Cation Dehydration by Surface‐Grafted Phenyl Groups for Enhanced C_2+_ Production in Cu‐Catalyzed Electrochemical CO_2_ Reduction,” Journal of the American Chemical Society 147, no. 37 (2025): 34001–34010, 10.1021/jacs.5c11313. 40919638 PMC12447509

[18] M. H. Hicks , W. Nie , A. E. Boehme , H. A. Atwater , T. Agapie , and J. C. Peters , “Electrochemical CO_2_ Reduction in Acidic Electrolytes: Spectroscopic Evidence for Local pH Gradients,” Journal of the American Chemical Society 146, no. 36 (2024): 25282–25289, 10.1021/jacs.4c09512. 39215715 PMC11403608

[19] M. H. Hicks , N. B. Watkins , S. Castro , T. Agapie , and J. C. Peters , “Cation Competition Experiments during Electrochemical CO_2_ Reduction as a Probe of the Film‐Modified Copper Microenvironment,” Journal of the American Chemical Society 147, no. 42 (2025): 38158–38168, 10.1021/jacs.5c10155. 41060281

[20] M. A. A. Mahbub , J. R. C. Junqueira , and X. Wang , “Dynamic Transformation of Functionalized Bismuth to Catalytically Active Surfaces for CO_2_ Reduction to Formate at High Current Densities,” Advanced Functional Materials 34, no. 3 (2024): 2307752, 10.1002/adfm.202307752.

[21] M. A. A. Mahbub , D. Das , X. Wang , G. Lu , M. Muhler , and W. Schuhmann , “Towards the Use of Low‐concentration CO_2_ Sources by Direct Selective Electrocatalytic Reduction,” Angewandte Chemie International Edition 64 (2024): 202419775, 10.1002/anie.202419775. 39714331

[22] Z. Zhang , D. Xi , Z. Ren , and J. Li , “A Carbon‐efficient Bicarbonate Electrolyzer,” Cell Reports Physical Science 4, no. 11 (2023): 101662, 10.1016/j.xcrp.2023.101662.

[23] T. Li , E. W. Lees , M. Goldman , D. A. Salvatore , D. M. Weekes , and C. P. Berlinguette , “Electrolytic Conversion of Bicarbonate into CO in a Flow Cell,” Joule 3, no. 6 (2019): 1487, 10.1016/j.joule.2019.05.021.

[24] T. Li , E. W. Lees , Z. Zhang , and C. P. Berlinguette , “Conversion of Bicarbonate to Formate in an Electrochemical Flow Reactor,” ACS Energy Letters 5, no. 8 (2020): 2624–2630, 10.1021/acsenergylett.0c01291.

[25] Y. C. Li , G. Lee , T. Yuan , et al., “CO_2_ Electroreduction from Carbonate Electrolyte,” ACS Energy Lett 4, no. 6 (2019): 1427, 10.1021/acsenergylett.9b00975.

[26] H. Ma , E. Ibáñez‐Alé , R. Ganganahalli , J. Pérez‐Ramírez , N. López , and B. S. Yeo , “Direct Electroreduction of Carbonate to Formate,” Journal of the American Chemical Society 145, no. 45 (2023): 24707, 10.1021/jacs.3c08079. 37924283 PMC10655187

[27] D. J. D. Pimlott , A. Jewlal , Y. Kim , and C. P. Berlinguette , “Oxygen‐Resistant CO_2_ Reduction Enabled by Electrolysis of Liquid Feedstocks,” Journal of the American Chemical Society 145, no. 48 (2023): 25933–25937, 10.1021/jacs.3c08930. 37983190

[28] H. Song , C. A. Fernández , H. Choi , P.‐W. Huang , J. Oh , and M. C. Hatzell , “Integrated Carbon Capture and CO Production from Bicarbonates through Bipolar Membrane Electrolysis,” Energy & Environmental Science 17, no. 10 (2024): 3570–3579, 10.1039/d4ee00048j.

[29] M. Dunwell , Q. Lu , and J. M. Heyes , “The Central Role of Bicarbonate in the Electrochemical Reduction of Carbon Dioxide on Gold,” Journal of the American Chemical Society 139, no. 10 (2017): 3774–3783, 10.1021/jacs.6b13287. 28211683

[30] A. Wuttig , Y. Yoon , J. Ryu , and Y. Surendranath , “Bicarbonate Is Not a General Acid in Au‐Catalyzed CO_2_ Electroreduction,” Journal of the American Chemical Society 139, no. 47 (2017): 17109–17113, 10.1021/jacs.7b08345. 28978199

[31] J. S. Zeng , N. Corbin , K. Williams , and K. Manthiram , “Kinetic Analysis on the Role of Bicarbonate in Carbon Dioxide Electroreduction at Immobilized Cobalt Phthalocyanine,” ACS Catal 10, no. 7 (2020): 4326, 10.1021/acscatal.9b05272.

[32] G. Marcandalli , M. Villalba , and M. T. M. Koper , “The Importance of Acid–Base Equilibria in Bicarbonate Electrolytes for CO_2_ Electrochemical Reduction and CO Reoxidation Studied on Au( hkl ) Electrodes,” Langmuir 37, no. 18 (2021): 5707–5716, 10.1021/acs.langmuir.1c00703. 33913319 PMC8154874

[33] G. Marcandalli , A. Goyal , and M. T. M. Koper , “Electrolyte Effects on the Faradaic Efficiency of CO_2_ Reduction to CO on a Gold Electrode,” ACS Catalysis 11, no. 9 (2021): 4936–4945, 10.1021/acscatal.1c00272. 34055454 PMC8154322

[34] G.‐H. Deng , Q. Zhu , J. Rebstock , T. Neves‐Garcia , and L. R. Baker , “Direct Observation of Bicarbonate and Water Reduction on Gold: Understanding the Potential Dependent Proton Source during Hydrogen Evolution,” Chemical Science 14, no. 17 (2023): 4523–4531, 10.1039/D3SC00897E. 37152268 PMC10155912

[35] L. Huang , G. Gao , J. Zhao , W. L. Roberts , and X. Lu , “Electrocatalytic Upcycling of High‐pressure Captured CO_2_ to Ethylene,” Nature Catalysis 8, no. 9 (2025): 968–976, 10.1038/s41929-025-01411-9.

[36] A. Rossen , N. Daems , D. Choukroun , and T. Breugelmans , “Differential Pressure across a Gas Diffusion Electrode Controls Efficiency of Liquid‐Fed Electrolyzers for CO_2_ Electroreduction at Elevated Temperatures,” ACS Sustainable Chemistry & Engineering 12, no. 20 (2024): 7935–7942, 10.1021/acssuschemeng.4c01908.

[37] P. Jeanty , C. Scherer , E. Magori , K. Wiesner‐Fleischer , O. Hinrichsen , and M. Fleischer , “Upscaling and Continuous Operation of Electrochemical CO_2_ to CO Conversion in Aqueous Solutions on Silver Gas Diffusion Electrodes,” Journal of CO2 Utilization 24 (2018): 454, 10.1016/j.jcou.2018.01.011.

[38] L. M. Baumgartner , A. Goryachev , and C. I. Koopman , “Electrowetting Limits Electrochemical CO_2_ Reduction in Carbon‐free Gas Diffusion Electrodes,” Energy Advances 2, no. 11 (2023): 1893–1904, 10.1039/D3YA00285C. 38013932 PMC10634457

[39] L. M. Baumgartner , C. I. Koopman , A. Forner‐Cuenca , and D. A. Vermaas , “Narrow Pressure Stability Window of Gas Diffusion Electrodes Limits the Scale‐Up of CO_2_ Electrolyzers,” ACS Sustainable Chemistry & Engineering 10, no. 14 (2022): 4683–4693, 10.1021/acssuschemeng.2c00195. 35433135 PMC9006256

[40] J. Ma , J. Yan , J. Xu , J. Ni , H. Zhang , and L. Lu , “Dynamic Ion Exchange Engineering BiOI‐derived Bi_2_O_2_CO_3_ to Promote CO_2_ Electroreduction for Efficient Formate Production,” Chemical Engineering Journal 455 (2023): 140926, 10.1016/j.cej.2022.140926.

[41] D. Yao , C. Tang , A. Vasileff , X. Zhi , Y. Jiao , and S.‐Z. Qiao , “The Controllable Reconstruction of Bi‐MOFs for Electrochemical CO 2 Reduction through Electrolyte and Potential Mediation,” Angewandte Chemie 133, no. 33 (2021): 18326–18332, 10.1002/ange.202104747. 34240788

[42] M. Sovago , R. K. Campen , G. W. H. Wurpel , M. Müller , H. J. Bakker , and M. Bonn , “Vibrational Response of Hydrogen‐bonded Interfacial Water Is Dominated by Intramolecular Coupling,” Physical Review Letters 100, no. 17 (2008): 173901, 10.1103/PhysRevLett.100.173901. 18518288

[43] T. Seki , K.‐Y. Chiang , and C.‐C. Yu , “The Bending Mode of Water: a Powerful Probe for Hydrogen Bond Structure of Aqueous Systems,” The Journal of Physical Chemistry Letters 11, no. 19 (2020): 8459, 10.1021/acs.jpclett.0c01259. 32931284 PMC7584361

[44] Y. Tian , B. Huang , and Y. Song , “Effect of Ion‐specific Water Structures at Metal Surfaces on Hydrogen Production,” Nature Communications 15, no. 1 (2024): 7834, 10.1038/s41467-024-52131-w. PMC11380671 39244565

[45] H. Zhang , J. Gao , D. Raciti , and A. S. Hall , “Promoting Cu‐catalysed CO_2_ Electroreduction to Multicarbon Products by Tuning the Activity of H_2_O,” Nature Catalysis 6, no. 9 (2023): 807, 10.1038/s41929-023-01010-6.

[46] H. Zhang , D. Raciti , and A. S. Hall , “Disordered Interfacial H2O Promotes Electrochemical C–C Coupling,” Nature Chemistry 17, no. 8 (2025): 1161–1168, 10.1038/s41557-025-01859-z. 40702128

[47] R. J. Gomes , R. Kumar , H. Fejzić , B. Sarkar , I. Roy , and C. V. Amanchukwu , “Modulating Water Hydrogen Bonding within a Non‐aqueous Environment Controls Its Reactivity in Electrochemical Transformations,” Nature Catalysis 7, no. 6 (2024): 689–701, 10.1038/s41929-024-01162-z.

[48] H. Fejzić , R. Kumar , R. J. Gomes , et al., “Water Clustering Modulates Activity and Enables Hydrogenated Product Formation during Carbon Monoxide Electroreduction in Aprotic Media,” Journal of the American Chemical Society 147, no. 22 (2025): 18445, 10.1021/jacs.4c07865. 40388344

[49] K. Lau , M. A. A. Mahbub , and N. Zhang , “Mechanistic Insights into the Competition between Electrochemical CO_2_ Reduction and Hydrogen Evolution on Ag‐based Electrocatalysts via Operando Raman Spectroscopy,” Chemical Science 16, no. 48 (2025): 23160, 10.1039/D5SC04774A. 41190192 PMC12581200

[50] W. Ye and W. Yang , “A Holistic View of Ions and Water Arrangements at the Electrified Cu Surface during Electrochemical CO_2_ Conversion,” The Journal of Physical Chemistry C 130, no. 1 (2026): 827–837, 10.1021/acs.jpcc.5c07614.

[51] W. Nie , G. P. Heim , N. B. Watkins , T. Agapie , and J. C. Peters , “Organic Additive‐Derived Films on Cu Electrodes Promote Electrochemical CO 2 Reduction to C 2+ Products under Strongly Acidic Conditions,” Angewandte Chemie International Edition 62, no. 12 (2023): 202216102, 10.1002/anie.202216102. 36656130

[52] P. A. Villaroel , E. Kecsenovity , and C. Janáky , “Local Variations in Current Density and Selectivity in CO_2_ Electrolyzers,” ACS Energy Lett 11, no. 2 (2026): 2029, 10.1021/acsenergylett.5c03770. 41710783 PMC12910950

