# Supplementary material for: Operando Raman Spectroscopy Reveals Backpressure‐Controlled Dynamics in Diluted CO2 Electrolysis
Source: Adv Sci (Weinh). 2026 Jun 9;13(49):e75908. doi: 10.1002/advs.75908 (PMC13336875; doi:10.1002/advs.75908)
Supplement: Supplementary file 1 [file ADVS-13-e75908-s001.docx]

Supporting Information

**Operando Raman Spectroscopy Reveals Backpressure-Controlled Dynamics in Diluted CO_2_ Electrolysis**

Muhammad Adib Abdillah Mahbub, Bashir Eid, Kinran Lau, Nini Zhang, Thomas Quast, Ann Cathrin Brix, Debanjan Das, and Wolfgang Schuhmann*

# Experimental

**Materials:** All chemicals were used without further purification. Bismuth (III) nitrate penta­hydrate (Bi(NO_3_)_3_∙5H_2_O, 98.0%), 2-methylimidazole (C_4_H_6_N_2_, 99%), hexadecyltrimethylam­monium bromide (CTAB - C_16_H_33_N(CH_3_)_3_Br, ≥98.0%), potassium bicarbonate (KHCO_3_, 99.7%), and poly(tetrafluoroethylene) (PTFE, 1 μm particle size) were from Sigma-Aldrich. Potassium hydroxide (KOH) was from Fisher Scientific. Deionised water was from a Millipore purification system (18.2 MΩ). The KOH electrolyte was purified using a Chelex® resin column. Gas diffusion layers (GDL) with microporous layers and hydrophobic treatment (H23C6, Freudenberg), and a Nafion 117 membrane (Merck) were used. Carbon dioxide (CO_2_), nitrogen (N_2_), and 2.25% CO_2_ with N_2_ balance gas bottles were from Air Liquide.

**Synthesis of Bi[2-MeIm]:** The Bi[2-MeIm] was synthesized following previously published procedures.^[1]^ 0.978 g of Bi(NO_3_)_3_∙5H_2_O and 15 mg CTAB were dispersed in 20 ml water (solution A). 4.5 g of 2-methylimidazole was dispersed in 80 ml water (solution B). Then, both solutions were mixed by pouring solution A into solution B under stirring to form a white milky solution. The solution was kept overnight while stirring, and the white precipitate was collected by centrifugation and washed once with ethanol after three times with water. The final Bi[2-MeIm] precipitate was dried in an oven at 70 °C overnight.

**Preparation of gas diffusion electrodes (GDE):** A catalyst/PTFE suspension was prepared by dispersing 2 mg of Bi[2-MeIm] and 1 mg of PTFE in 1.5 ml isopropanol, followed by sonication for 30 min (frequency 37 kHz). The suspension was drop-cast onto H23C6 carbon paper (diameter 18 mm) positioned on a frit connected to a vacuum pump, obtaining a final loading of approximately 1 mg cm^-2^. The catalyst-modified GDE was dried in air and attached to a 3D printed gasket using Cu tape, which also acts as a current collector.

**CO_2_ electrolysis experiments:** CO_2_ reduction reaction (eCO_2_RR) measurements were carried out in a three-electrode flow-through electrolyzer cell using an Autolab PGSTAT302N poten­tiostat/galvanostat connected online to a gas chromatograph (GC, SRI instrument) equipped with a thermal conductivity detector (TCD) to quantify H_2_ and a flame ionization detector methanizer (FID_meth_) to quantify CO. The carrier gas was N_2_ and the column temperature was 90 °C. During the measurement, the gaseous products were from the headspace in the catholyte compartment as the top injection and from the gas feed compartment as the bottom injection. Both gas volumes were combined with a Y-connector and to a bubble counter before entering the GC. For the electrolysis cell, the cathode/working electrode (WE) was the GDE, the reference electrode (RE) was Ag/AgCl (3 M KCl), and the anode/counter electrode (CE) was Ni foam. The WE and CE compartment was separated by a Nafion 117 as a cation exchange membrane with 1 M KHCO_3_ as catholyte and 1 M KOH as anolyte circulated through the cell using a Perimax 12 peristaltic pump.

We performed eCO_2_RR at 20% and 5% CO_2_ concentration, with N_2_ as dilutant to balance the concentration. The total flow rate of the binary mixed gas was kept constant at 20 ml min^-1^. Galvanostatic measurements were performed at -100 mA cm^-2^ for the eCO_2_RR at 20% CO_2_, and lower current densities for 5% CO_2_, normalized to the geometrical area of the GDE of 0.95 cm^2^, to derive product selectivity. The measurements started with a galvanodynamic linear sweep from 0 to 25 mA cm^-2^ for catalyst pre-reduction, followed by chronopotentiometry (CP) measurements and galvanostatic electrochemical impedance spectroscopy (GEIS) measure­ments every after CP. eCO_2_RR experiments with more diluted CO_2_ concentrations (2.25%) were performed by feeding the gas compartment directly from 2.25% CO_2_ (N_2_ balance) gas bottles. Various backpressures were applied to the gas compartment using a water column for +2 mbar and +15 mbar, and a digital backpressure controller (Alicat) for +100 mbar experiments.

The potentials were converted to the reversible hydrogen electrode (RHE) with iR correction using equation 1:

$V_{RHE}=[V_{Ag/AgCl (3 M KCl)}+0.210+(0.059\times pH)]-iR$	1)

with $V_{RHE}$ is the calculated potential versus RHE, $V_{Ag/AgCl (3 M KCl)}$ is the potential versus reference electrode of Ag/AgCl (3 M KCl), and $iR$ is obtained from multiplying applied current by the uncompensated resistance (solution resistance) from the GEIS measurements.

The Faradaic efficiency (FE) for gas product was calculated using equation 2:

${FE}_{a}=\frac{n_{a}z_{a}fF}{VI_{t}}$	2)

where *n_a_* is the concentration of the product “a” (ppm from the GC, converted into vol% by multiplying with 10^-6^), *z_a_* is the number of electrons required to generate the products (two for carbon monoxide and hydrogen), *f* is the gas flow rate in L s^-1^, *F* is the Faraday constant (96485 C mol^-1^), *V* is the molar volume of an ideal gas at 25 ^o^C (24.456 L mol^-1^), and *I_t_* is the total current in A.

The liquid products were analyzed by high-performance liquid chromatography (HPLC, Dionex ICS-5000 ThermoFisher) with an ion-exclusion column (Aminex HPX-87H; Bio-Rad), a diode array detector at a wavelength of 220 nm, and a refractive index (RI) detector (RefractoMax520). Formate was analyzed using the RI detector. The eluent was 4 mM H_2_SO_4_ with a flow rate of 0.6 mL min^-1^, and a column temperature of 70 °C. The sample was prepared by mixing 440 µL of collected liquid product with 110 µL of 2.5 M H_2_SO_4_ for acidification. The FE for the liquid products was determined using equation 3:

${FE}_{b}=\frac{n_{b}z_{b}F}{I_{t}. t}\times100\%$	3)

*n_b_* is the calculated number of mols of product “b”, *z_b_* is the electron transfer number, and *t* is the measurement duration.

**Operando Raman spectroelectrochemistry:** Spectroelectrochemical Raman measurements were performed on a Lab-RAM HR Raman microscopy system (Horiba Jobin Yvon HR550) with a 532 nm laser, a water immersion objective (Olympus LUMFL, 60x, numerical aperture = 1.10), a monochromator (grating of 1800 grooves/mm), and a Synapse CCD detector. The measurements were carried out in a customized 3D-printed operando Raman cell with a gas compartment, and anode and cathode compartments separated with a membrane (**Figure S4**). A working electrode (catalyst-coated GDE), reference electrode (Ag/AgCl (3 M KCl)), counter electrode (Ni foam), 1 M KHCO_3_ and 1 M KOH as the catholyte and anolyte, respectively, were used for consistency with the electrolyzer experiments. A Gamry REF600 potentiostat was used for the electrochemical measurements.

The measurement process started by calibrating the Rayleigh scattering, and the calibration was confirmed using the spectrum of a Si wafer at around 520 cm^-1^. Raman spectra were then recorded without electrolyte, with the objective lens adjusted to the appropriate working distance to ensure a sharp focus and strong signal. After introducing the electrolyte, the objective lens was re-focused to maintain high spectral intensity. Once focused, potential-dependent measurements were conducted without any further realignment. The Raman spectra acquisition was done for 20 s over 2 scans in the range of 50-3900 cm^-1^. During the potential-dependent experiments, Raman spectra were collected after 15 s of the respective applied potential. For the spectral analysis, the raw spectra were first smoothed using a Savitzky-Golay filter with a window size of 50 and a polynomial order of 3. Baseline subtraction was then performed using the automated Asymmetric Least Squares (ALS) smoothing of the baseline using an asymmetric factor (0), threshold (0.005), smoothing factor (8), and number of iterations (10). The resulting spectra were normalized to a reference peak at 95-100 cm^-1^ as a characteristic band of the instrument.

**(Bi)carbonate buffer chemistry and eCO_2_RR:** In the eCO_2_RR system, the dominant charge carriers are CO_3_^2-^ and HCO_3_^-^. OH^-^ from the electrolyte (in alkaline systems) or generated at the cathode during eCO_2_RR and HER, immediately reacts with CO_2_ to form (bi)carbonates. Thus, (bi)carbonate buffer chemistry is a critical descriptor for CO_2_ availability because it acts as a dynamic CO_2_ species and controls the local pH. The HER half-reaction involves 2 e^-^ transfer and yields 2 mols of OH^-^ during electrolysis, whereas CO_2_-to-HCOO^-^ produces 1 mol of OH^-^ per 2 mols of e^-^ transferred. Therefore, it is assumed that CO_2_ neutralization due to the reaction of CO_2_ with OH^-^, resulting in CO_3_^2-^/HCO_3_^-^ species in formate-selective CO_2_ electrolysis is lower than in a HER-dominant system. The bicarbonate buffer system, as well as eCO_2_RR to formate and HER involve the following reactions:

${CO}_{2}+{OH}^{-}\rightleftharpoons{{HCO}_{3}}^{-}$	4)

${{HCO}_{3}}^{-}+{OH}^{-}\rightleftharpoons{{CO}_{3}}^{2-}+H_{2}O$	5)

${CO}_{2}+{2e}^{-}+H_{2}O\rightleftharpoons{HCOO}^{-}+{OH}^{-}$	6)

${2H}_{2}O+{2e}^{-}\rightleftharpoons H_{2}+{2OH}^{-}$	7)

and continuous CO_2_ supply from the backside of the GDE together with high backpressure might shift the equilibrium of the (bi)carbonate electrolyte system and maintain the dissolved CO_2_ being available as an active reactant for eCO_2_RR,

${CO}_{2}+H_{2}O\rightleftharpoons{{HCO}_{3}}^{-}+H^{+}$	8)

${{CO}_{3}}^{2-}+H^{+}\rightleftharpoons{{HCO}_{3}}^{-}$	9)

**Figure S1**. Recorded potential vs. Ag/AgCl (3 M KCl) as a function of time at a constant current density of -100 mA cm^-2^ (normalized to the geometric area, A = 0.95 cm^2^) for a eCO_2_RR experiment at 20% CO_2_ concentration.

**Figure S2**. a) Schematic experimental setup for bicarbonate electrolysis. Electrochemical performance including the b) recorded potential, c) corresponding Nyquist plot, d) calculated corrected potential, e) Faradaic efficiency for formate as a function of current densities, and f) HPLC chromatogram.

**Figure S3**. a) Calculated corrected potential as a function of current density. b) Faradaic efficiency from the experiment using the Bi-based catalyst at 100% CO_2_ concentration.

**Figure S4**. 3D-printed GDE-based Raman cell including its cross-section for spectroelectro­chemical measurements comprised of catholyte and anolyte compartments separated by a Nafion membrane (cathode exchange membrane, CEM). It features a Ni foam counter electrode (CE), a Ag/AgCl (3 M KCl) reference electrode (RE), and the gas diffusion electrode (GDE) as the working electrode (WE).

**Figure S5**. Raman spectra of Bi[2-MeIm] with and without electrolyte.

**Figure S6**. Raw Raman spectra at +2 and +15 mbar backpressure with their corresponding baselines for subtraction. Potentials are corrected and converted to RHE.

**Figure S7**. a) Normalized Raman spectra of Bi[2-MeIm]. b) Enlarged bicarbonate and carbonate Raman signals at different corrected potentials versus RHE.

**Figure S8**. Electrochemical performance during spectroelectrochemical measurements for +2 and +15 mbar backpressure, including the recorded potentials, the corresponding Nyquist plots, and the potential-current density plot.

**Figure S9**. Raman spectra and the electrochemical performance during the spectroelectroche­mical measurements for +2 and +20 mbar backpressure, including recorded potentials, corres­ponding Nyquist plots, and the enlarged bicarbonate and carbonate Raman signals at different applied currents.

**Figure S10**. a). Current density-corrected potential plot from spectroelectrochemical experi­ments. b) Quantitative analysis of the carbonate-bicarbonate ratio as a function of current density.

Figure S11. Control experiments using the Bi-based catalyst in different electrolyte solutions (same cations) to observe the overlapping signals from formate and bicarbonate species.

**Figure S12**. Raman spectra for +2 and +15 mbar backpressure with and without electrolyte. After spectral processing, the overall O-H stretching vibration mode has similar intensities for better comparison after potential-dependent spectroelectrochemical measurements.

**Figure S13**. a) Normalized and deconvoluted Raman spectra. b) Water percentage and the corresponding peak I/peak II ratio, derived from the O-H stretching vibration signal at various applied potentials at +2 mbar backpressure. Potentials given are versus Ag/AgCl (3 M KCl).

**Figure S14**. Spectroelectrochemical reproducibility measurements at different backpressures, including normalized Raman spectra, enlarged (bi)carbonate signals, water (O-H) stretching vibration mode, the recorded potentials given are versus Ag/AgCl (3 M KCl), and the Nyquist plots from the electrochemical measurements.

**Figure S15**. Electrochemical potential-current density plot for a) potential vs. Ag/AgCl (3 M KCl), and b) potential vs. RHE. c) Quantitative analysis of the carbonate-bicarbonate ratio, and d) water (O-H) stretching band area at +2 and +15 mbar backpressure as a function of applied potentials.

# References for Supplementary Information:

[1]	M. A. A. Mahbub, J. R. C. Junqueira, X. Wang, et al. “Dynamic Transformation of Functionalized Bismuth to Catalytically Active Surfaces for CO_2_ Reduction to Formate at High Current Densities,” *Adv. Funct. Mater.* (2024), *34* , 2307752. 10.1002/adfm.202307752.
